# Genome-Wide Identification of Detoxification Genes in Wild Silkworm *Antheraea pernyi* and Transcriptional Response to Coumaphos

**DOI:** 10.3390/ijms24119775

**Published:** 2023-06-05

**Authors:** Dong-Bin Chen, Run-Xi Xia, Qun Li, Yu-Ping Li, Hui-Ying Cao, Yan-Qun Liu

**Affiliations:** College of Bioscience and Biotechnology, Shenyang Agricultural University, 120 Dongling Road, Shenyang 110866, China; 18302468026@163.com (D.-B.C.); xiarunxi@126.com (R.-X.X.);

**Keywords:** *Antheraea pernyi*, detoxification gene, coumaphos, differentially expressed genes

## Abstract

For a half-century, the commercial wild silkworm, *Antheraea pernyi*, has been protected by coumaphos, which is an internal organophosphorus insecticide used to kill the potential parasitic fly larvae inside. Knowledge about the detoxification genes of *A. pernyi* as well as the detoxification mechanism for this species remains severely limited. In this study, we identified 281 detoxification genes (32 GSTs, 48 ABCs, 104 CYPs, and 97 COEs) in the genome of this insect, which are unevenly distributed over 46 chromosomes. When compared to the domesticated silkworm, *Bombyx mori*, a lepidopteran model species, *A. pernyi* has a similar number of ABCs, but a greater number of GSTs, CYPs, and COEs. By transcriptome-based expression analysis, we found that coumaphos at a safe concentration level significantly changed the pathways related to ATPase complex function and the transporter complex in *A. pernyi*. KEGG functional enrichment analysis indicated that protein processing in the endoplasmic reticulum was the most affected pathway after coumaphos treatment. Finally, we identified four significantly up-regulated detoxification genes (*ABCB1*, *ABCB3*, *ABCG11*, and *ae43*) and one significantly down-regulated detoxification gene (*CYP6AE9*) in response to coumaphos treatment, suggesting that these five genes may contribute to detoxification of coumaphos in *A. pernyi*. Our study provides the first set of detoxification genes for wild silkworms from Saturniidae and highlights the importance of detoxification gene repertoire in insect pesticide tolerance.

## 1. Introduction

Chinese oak silkworm, *Antheraea pernyi* Guérin-Méneville 1855 (Lepidoptera: Saturniidae), is one of the most well-known wild silkworms, and it still feeds on oak trees (*Quercus*) during the whole larval stage [1,2,3]. Commercial rearing of *A. pernyi* to produce raw silk as fiber has a long history and is practiced in China, India, Japan, and Korea [1]. This significant commercial insect is also employed as a source of insect food (larva, pupa, and moths) for cosmetics and human use. Due to its vivid larval body, *A. pernyi* serves as a significant source of tourism in addition to its commercial applications. In investigations of breeding technique, development, metabolism regulation, and immune response, this species has also served as a well-known experimental model system [4,5,6].

The parasitic fly, *Blepharipa tibialis* Chao (Diptera: Tachinidae), is an important economic pest that harms the larvae of *A. pernyi*. The damage rate by *B. tibialis* ranged from 20% to 70% in spring in the Liaoning production area (North China) [7]. In production practice, coumaphos (CAS No. 56-72-4), also known as Miecanying (Chinese trade name), has been selected as an internal organophosphorus insecticide that can kill the potential larvae of the parasitic flies inside the silkworm and save the larvae of *A. pernyi* at the safety concentration [8]. Coumaphos works by preventing the decomposition of acetylcholinesterase in insects, obstructing nerve transmission, and killing the insects [9,10,11]. The treatment of coumaphos at a higher concentration level has also neurotoxic effects on *A. pernyi*, which can result in poisoning symptoms, such as spewing gastric juice and refusing to climb trees [9]. Lepidopteran insects including numerous insect pests have evolved a great tolerance to insecticides [10,11,12,13]. The genes for glutathione S-transferase cytochrome (GST; EC 2.5.1.18), ATP-binding cassette transporter (ABC transporter), cytochrome P450 monooxygenase (CYP/P450), and carboxylesterase (COE; EC 3.1.1.1) are among those that are frequently more abundant in butterflies and moths. Numerous detoxification genes, including GSTs, ABC transporters, CYPs, and COEs, have been widely identified and functionally shown in Lepidoptera species as a result of the expansion of their transcriptomes and genomes [10,14,15,16,17,18]. However, little is known about the detoxification genes of *A. pernyi* as well as the detoxification mechanism for this species.

In this study, we present the identification and genomic analysis of the detoxification genes of *A. pernyi* using the first high-quality chromosome-scale assembled genome sequence [19]. This work provides a detailed analysis of the detoxification genes in the Chinese oak silkworm. We also used the transcriptomics approach to understand how coumaphos might change the gene expression patterns of the fat body of *A. pernyi* larvae and be linked with detoxification. The insect fat body is a loosely organized tissue that senses and monitors the timely delivery of nutrients to the insect body [20]. This tissue is a crucial component of insect metabolism, playing a role in immune regulation, growth and development, and metabolic detoxification [21]. The findings presented here may provide a better understanding of the molecular processes that contribute to antioxidation and detoxification during Chinese oak silkworm development following insecticide treatment. 

## 2. Results

### 2.1. Genome-Wide Identification of Detoxification Genes in A. pernyi

Using the amino acid sequences of the domestic silkworm *Bombyx mori* detoxification genes [14] as queries, we identified 281 candidate detoxification genes, including 32 GST, 48 ABC, 104 CYP, and 97 COE genes, by performing a local TBLASTN and HMMER search against the genome sequence of *A. pernyi* [19]. Table 1 lists the four detoxification-related gene families for two silkworms, *A. pernyi* and *B. mori* [14], and four nocturnal lepidopteran pests, *Spodoptera litura* [10], *Spodoptera frugiperda* (corn and rice strains) [12], *Helicoverpa armigera*, and *Helicoverpa zea* [11]. We found that *A. pernyi* has a higher number of detoxification genes than *B. mori*, except for the ABC gene family. In addition, when comparing the number of detoxification genes, the ABC genes for *S. frugiperda* were not identified. This leads us to conclude that *A. pernyi* and *B. mori* have fewer detoxification genes than the four lepidopteran pests listed above. In Supplemental Table S1, the specific details of the identified genes are presented, including gene name, position on the genome, transcription direction, number of exons, size of amino acids, the theoretical isoelectric point (pI), molecular weight (Mw), and subcellular localization. Each subfamily gene was given a name based on where it is located on the chromosomes. 

**Figure 1:**
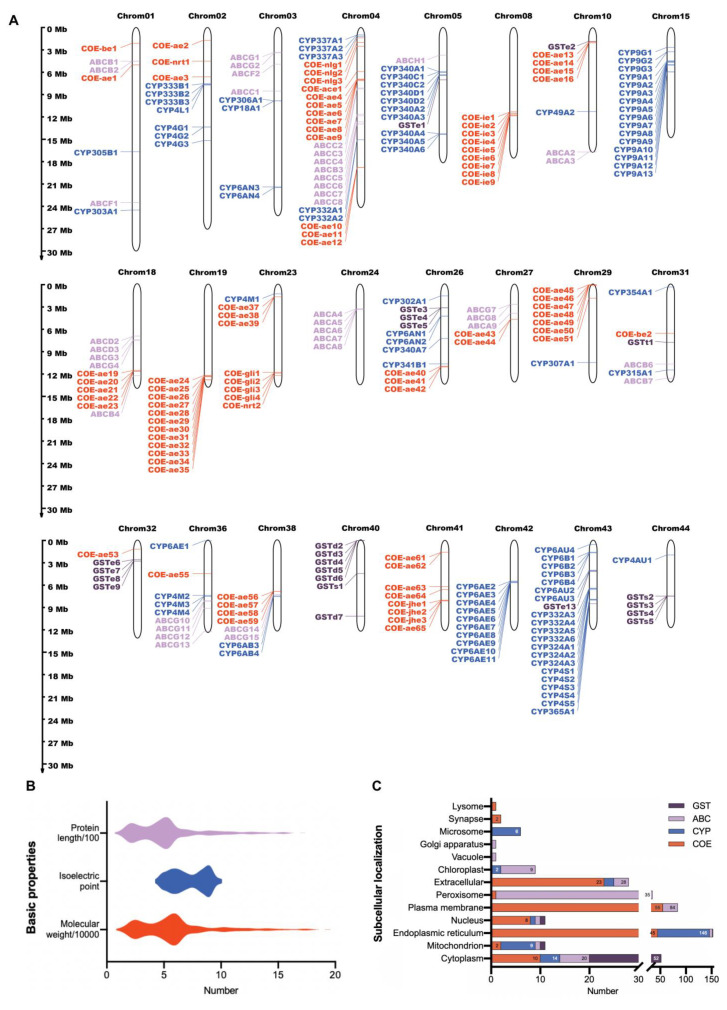
Identification and statistical analysis of the basic physicochemical characteristics of detoxification gene family members in *A. pernyi*. (**A**) The chromosomal locations of detoxification genes in the *A. pernyi* genome. The length of each chromosome is drawn to scale. Each family of detoxification genes is distinguished by a different color. Only those genes involved in five or more detoxification genes on the same chromosomes are shown. (**B**) Protein length, isoelectric point, and molecular mass of *A. pernyi* detoxification genes. (**C**) Subcellular localization of detoxification genes in *A. pernyi*.

We visualized the distribution of detoxification genes on 49 chromosomes of *A. pernyi* to better understand how they are distributed across the genome. Each chromosome has detoxification genes, except for chromosomes 13, 14, and 17. The chromosomes involved in five or more detoxification genes are depicted in Figure 1A. Examination of the chromosomal locations of silkworm detoxification genes showed that these were dispersed irregularly across the genome. For example, six of the seven genes in the delta subfamily of GSTs were located on chromosome 40; more than half of the known CYPs were located on chromosomes 5, 15, 42, and 43, which include 10, 16, 10, and 20 genes, respectively. The basic characteristics of different detoxification proteins varied greatly (Table S1). Most (76/281) were 300−400 amino acids, and the molecular mass was generally 30−40 kDa. Isoelectric point (pI) had a wide span, ranging from 4.23 to 9.99 (Figure 1B). Subcellular localization analysis showed that detoxification proteins were mainly distributed in the endoplasmic reticulum, plasma membrane, cytoplasm, and peroxisome (Figure 1C).

### 2.2. Glutathione S-Transferase (GST) Genes 

**Figure 2:**
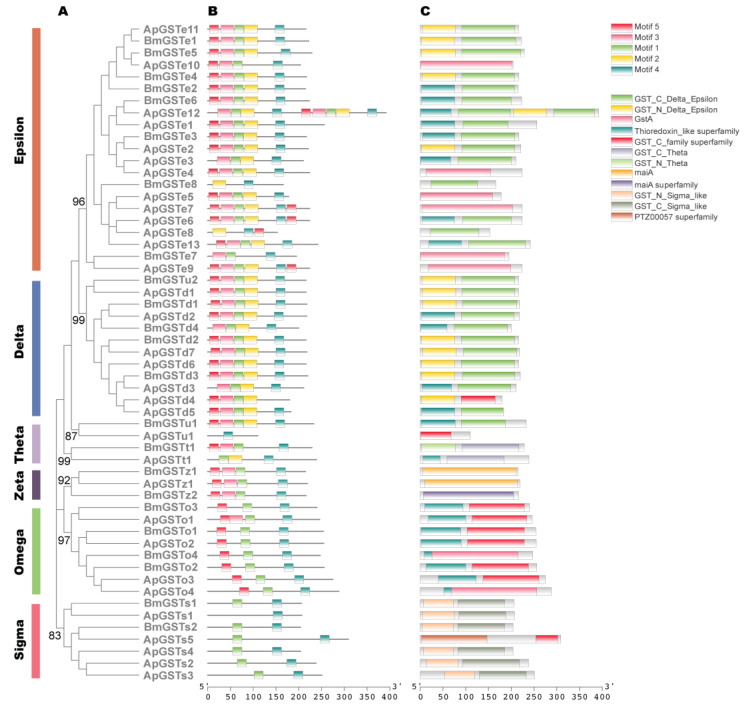
The phylogenetic and structural analysis of GST members. (**A**) The phylogenetic tree was constructed with the amino acid sequence of GSTs from *A. pernyi* (ApGSTs) and *B. mori* (BmGSTs). The numbers close to the nodes specify the bootstrap value for the clades. (**B**) The motif structure of ApGSTs and BmGSTs. (**C**) The domain structure of ApGSTs and BmGSTs.

The GSTs are covalently attaching enzymes that improve the solubility of compounds and aid in their excretion by covalently joining tiny, endogenous hydrophilic molecules [22]. Based on their subcellular localizations, GSTs have been divided into three classes: cytosolic, microsomal, and mitochondrial GSTs [15]. Among them, insect cytosolic GSTs have been classified into six clades, with the first two being unique and insect-specific: delta (δ), epsilon (*ε*), omega (ω), sigma (σ), theta (θ), and zeta (ζ) [23]. A total of 32 GST genes, which encoded proteins ranging in length from 110 to 392 amino acids, were identified in the genome of *A. pernyi* (Table S1). Based on the phylogenetic analysis with *B. mori* GST genes, the 32 *A. pernyi* GST genes were also divided into six various cytosolic classes: 7 in delta, 13 in epsilon, 4 in omega, 5 in sigma, 1 in theta, and 1 in zeta class; one gene that could not be easily assigned within one of the known classes was labeled as “unclassified” (Figure 2A; Table S1). We deduced from the phylogenetic tree that this unclassified gene is most closely related to the delta class, followed by the theta class. Compared with the GST genes of *B. mori* (n = 23), those of *A. pernyi* (n = 32) expanded. These expanded GST genes came from delta (n = 3), epsilon (n = 5), and sigma (n = 3). 

By determining the conserved motifs and domains, we found five conserved motifs, considered Motifs 1–5, in all of the GST proteins of *A. pernyi* (ApGSTs in the following). Most delta class and epsilon class branches had Motifs 1–5, while the zeta class did not have Motif 2. Most omega class branches had Motif 1, Motif 4, and Motif 5; most sigma class branches only had Motif 4 and Motif 5 (Figure 2A,B). Although the composition and number of motifs differed among different proteins, the motifs within most classes were similar. In addition, we discovered a total of 12 domains in ApGSTs, which are GST_C_Delta_Epsilon, GST_N_Delta_Epsilon, GstA, Thioredoxin_like superfamily, GST_C_family superfamily, GST_C_Theta, GST_N_Theta, maiA, maiA subfamily, GST_N_Sigma_like, GST_C_Sigma_like, and PTZ00057 superfamily (Figure 2C). At least one of the aforementioned domains was present in each ApGST. This further demonstrated that the GST family memberships were discovered in this investigation. In summary, similar conserved motifs and functional domains in the same classes forcefully back up phylogenetic analysis for classification.

### 2.3. ABC Transporter Genes 

**Figure 3:**
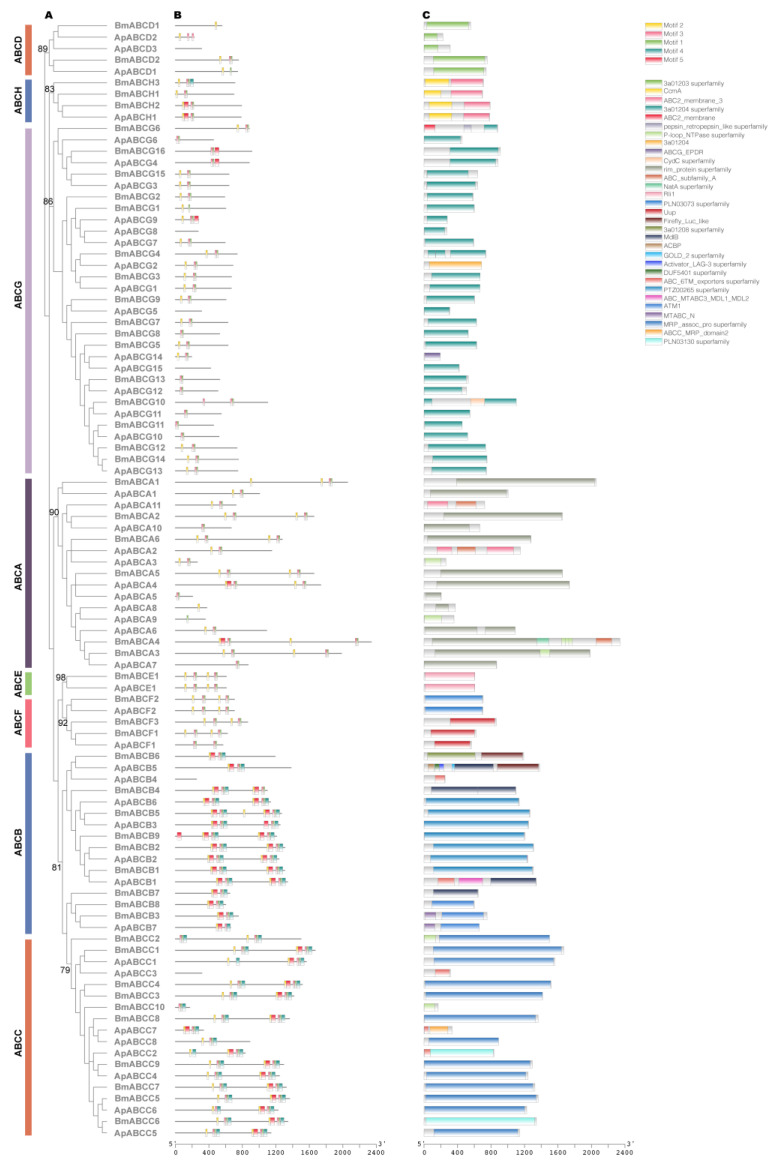
The phylogenetic and structural analysis of ABC members. (**A**) The phylogenetic tree was constructed with the amino acid sequence of ABCs from *A. pernyi* (ApABCs) and *B. mori* (BmABCs). The numbers close to the nodes specify the bootstrap value for the subfamilies. (**B**) The motif structure of ApABCs and BmABCs. (**C**) The domain structure of ApABCs and BmABCs.

The ABC transporters are among the largest families of transmembrane proteins [24]. Based on the similarity of ATP-binding site sequences, eukaryotic ABC transporters may be divided into eight subfamilies (ABCA-ABCH), seven of which (ABCA-ABCG) are presented in the human genome. The genome of the fruit fly, *Drosophila melanogaster*, contains the first definition of the eighth subfamily (ABCH) [24]. A total of 48 ABC transporter genes were identified in the *A. pernyi* genome. Based on the phylogenetic analysis with *B. mori* ABC transporter genes, the 48 *A. pernyi* ABC transporters (ApABCs in the following) were grouped into eight subfamilies (A-H): 11 in the A subfamily, 7 in the B subfamily, 8 in the C subfamily, 3 in the D subfamily, 1 in the E subfamily, 2 in the F subfamily, 15 in the G subfamily, and 1 in the H subfamily (Figure 3A; Table S1). The number of ABC transporter genes in *A. pernyi* (n = 48) was comparable to that in *B. mori* (n = 52). The compression of the ABC transporter gene number came from ABCB, ABCC, ABCF, ABCG, and ABCH subfamilies, whereas the expansion mainly occurred in the ABCA subfamily (*A. pernyi* (n = 11) vs. *B. mori* (n = 7)).

The five conserved motifs were identified in ApABCs and referred to as Motifs 1–5 (Figure 3B; Table S1). In addition, we discovered a total of 31 domains in all of the ApABC transporter proteins as we investigated the conserved domains and motifs in ApABCs, such as ABC_MTABC3_MDL1_MDL2, ABC_6TM_exporters superfamily, ABC2_membrane_3, and ABC_subfamily_A (Figure 3C). The composition and order of the conserved motifs and domains within every subfamily were similar. For example, the motifs of most members of the ABCB and ABCC subfamilies showed the following arrangement: Motif 2–Motif 5–Motif 3–Motif 1–Motif 4.

### 2.4. Cytochrome P450 (CYP) Genes

**Figure 4:**
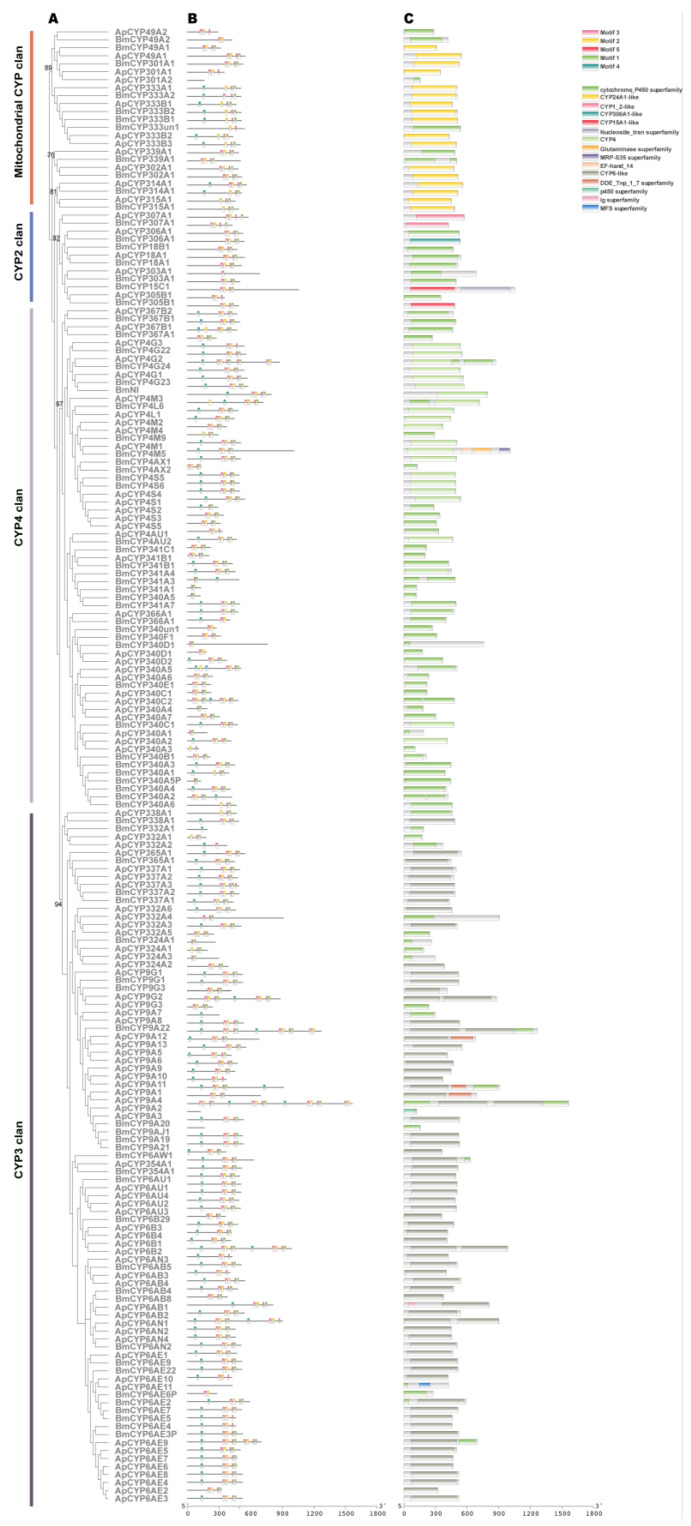
The phylogenetic and structural analysis of CYP members. (**A**) The phylogenetic tree was constructed with the amino acid sequence of CYPs from *A. pernyi* (ApCYPs) and *B. mori* (BmCYPs). The numbers close to the nodes specify the bootstrap value for the subfamilies. (**B**) The motif structure of ApCYPs and BmCYPs. (**C**) The domain structure of ApCYPs and BmCYPs.

The CYPs carry out the oxidative metabolism of several endogenous and exogenous substances. Before the class Insecta diverged, there were four large clades of insect CYPs that are represented by the CYP family in vertebrates as well: CYP2, CYP3, CYP4, and the mitochondrial (Mito) CYP clades [25]. The members of each clade share over 40% amino acid (AA) identities [26,27]. In the genome of *A. pernyi*, a total of 104 functional CYPs were identified and described. Based on the blast results of NCBI and the phylogenetic analysis with *B. mori* CYPs, these genes were classified into four clades: 5 in CYP2 Clade, 57 in CYP3 Clade, 30 in CYP4 Clade, and 12 in Mito Clade (Figure 4A; Table S1). The total number of *A. pernyi* CYP genes (n = 104) is larger than that of *B. mori* (n = 83). The expansion of CYP genes has occurred in Clade 3 (*A. pernyi* (n = 57) vs. *B. mori* (n = 31)) while the compression has occurred in Clade 2 (*A. pernyi* (n = 5) vs. *B. mori* (n = 7)) and Clade 4 (*A. pernyi* (n = 30) vs. *B. mori* (n = 34)). 

We further identified the conserved motifs and domains in CYPs of *A. pernyi*. MEME analysis [28] identified five conserved motifs in *A. pernyi* CYPs, which were referred to as Motifs 1–5 (Figure 4B). In Figure S1, the specific details of the conserved motifs are provided. It was found that 92 of the total 183 silkworm CYPs had Motifs 1–5 at the same time. We discovered that only 15 domains (such as CYP6-like, CYP4, CYP450 superfamily, CYP24A1-like, and CYP1_2-like) were shared by all *A. pernyi* CYP proteins (Figure 4B). Every gene that was found contained at least one domain, further proving that all 104 genes were members of the CYP family. In other words, functional domains and conserved motifs within a single subfamily strongly support subfamily classifications based on phylogenetic analysis. 

### 2.5. Carboxylesterase (COE) Genes

**Figure 5:**
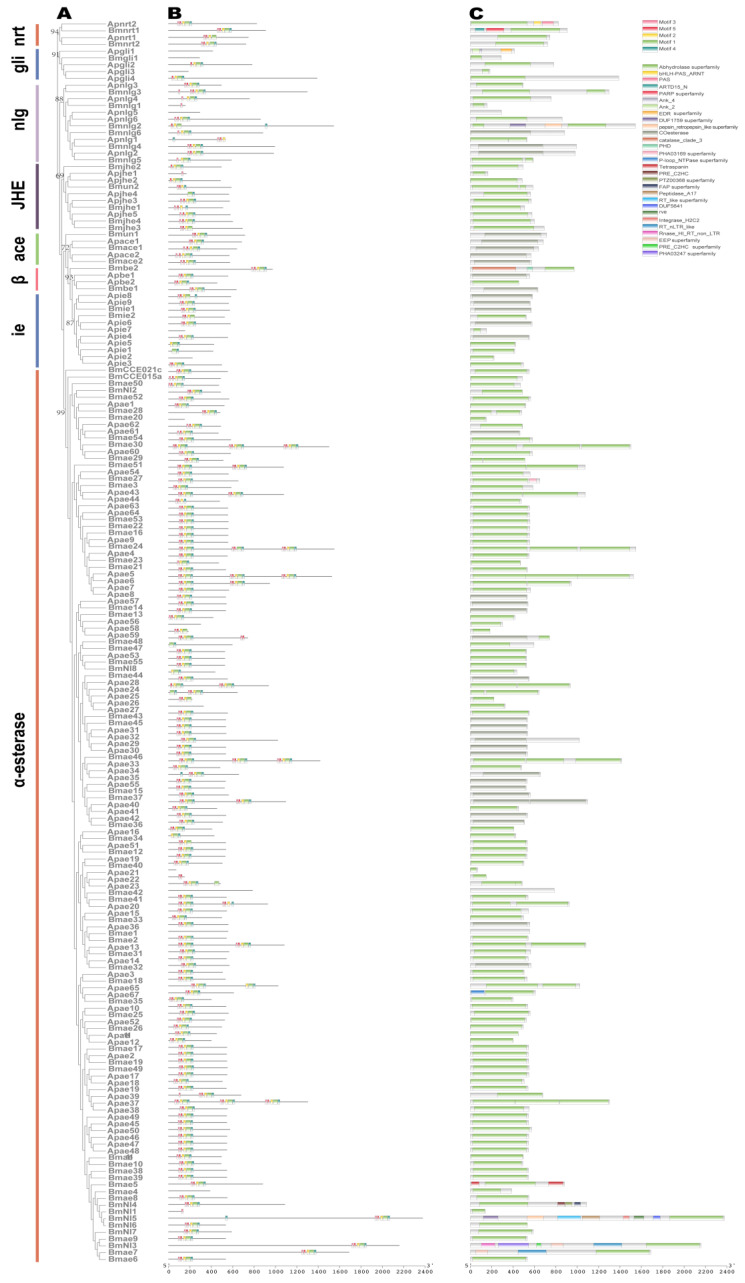
The phylogenetic and structural analysis of COE members. (**A**) The phylogenetic tree was constructed with the amino acid sequence of COEs from *A. pernyi* (ApCOEs) and *B. mori* (BmCOEs). The numbers close to the nodes specify the bootstrap value for the subfamilies. (**B**) The motif structure of ApCOEs and BmCOEs. (**C**) The domain structure of ApCOEs and BmCOEs.

The COEs are mainly attributed to B esterases, which were virtually irreversibly inhibited by organophosphate insecticides (OPs) [29,30,31]. Based on sequence similarity and substrate specificity, the insect COE gene sequences were divided into eight subfamilies, namely α-esterase (ae), β-esterase (be), juvenile hormone esterase (jhe), integument esterase (ie), gliotactin (gli), acetylcholinesterase (ace, AChE), neurotactin (nrt), and neuroligin (nlg) class [32]. A total of 97 functional COE genes were identified and characterized in the *A. pernyi* genome. These genes can be divided into the eight subfamilies listed: 67 in the ae, 2 in the be, 5 in the jhe, 9 in the ie, 4 in the gli, 2 in the AChE, 2 in the nrt, and 6 in the nlg subfamily (Table S1). The results of the phylogenetic analysis of the COE genes from *A. pernyi* and *B. mori* are displayed in Figure 5A. The number of COEs in the *A. pernyi* genome was expanded to the 87 COE genes recently reported in *B. mori* [14]. The expansion of COE genes has occurred in ae subfamily (*A. pernyi* (n = 67) vs. *B. mori* (n = 55)), gli subfamily (*A. pernyi* (n = 4) vs. *B. mori* (n = 1)), and ie subfamily (*A. pernyi* (n = 9) vs. *B. mori* (n = 2)). We found that gene duplication events have occurred in the ae subfamily of COE genes after the divergence of the two silkworms; examples include Apae45-50 and Bmae4-11. 

In general, five conserved motifs were found in the COE proteins of *A. pernyi* (ApCOE in the following) and considered Motifs 1–5 (Figure 5B and Figure S1). We discovered that most ApCOE proteins contained at least one conserved motif. The order of these motifs was mostly Motif 3–Motif 5–Motif 1–Motif 4–Motif 2. We discovered a total of 29 domains in ApCOEs to further determine the functional domains in ApCOEs (Figure 5C). Most of ApCOEs had domains from the Abhydrolase superfamily or COesterase, proving that the proteins found are the COE gene family members. 

### 2.6. Expression Patterns of Detoxification Genes Associated with Coumaphos Stress

Lepidopteran insects include numerous insect pests that have evolved a significant resistance to insecticides [10,12]. Chinese oak silkworm, which lives in the field during the whole larval stage and may have more resilience to hardship, is distinct from the domestic model insect *B. mori*. We performed a transcriptome study on the fat body of larvae that had been exposed to coumaphos to comprehend how important detoxification genes of *A. pernyi* respond to coumaphos. Transcriptomic sequencing data were generated utilizing 16 fat body cDNA libraries (coumaphos/ultrapure water treatment). After eliminating adapters, ambiguous nucleotides, and low-quality sequences, we obtained 36,163,814–46,894,904 clean reads from the fat body transcriptomes of *A. pernyi*. Clean sequencing data spanning 5.71–6.61 Gbp were produced with a Q30 range of 94.13–94.78%. An overview of the sequencing and assembly process is presented in Supplemental Table S2. The sequence information for *A. pernyi* fat bodies has been deposited in the NCBI Sequence Read Archive (SRA) database under accession numbers SRR21609102-117 and is linked to the Bioproject PRJNA879970. 

**Figure 6:**
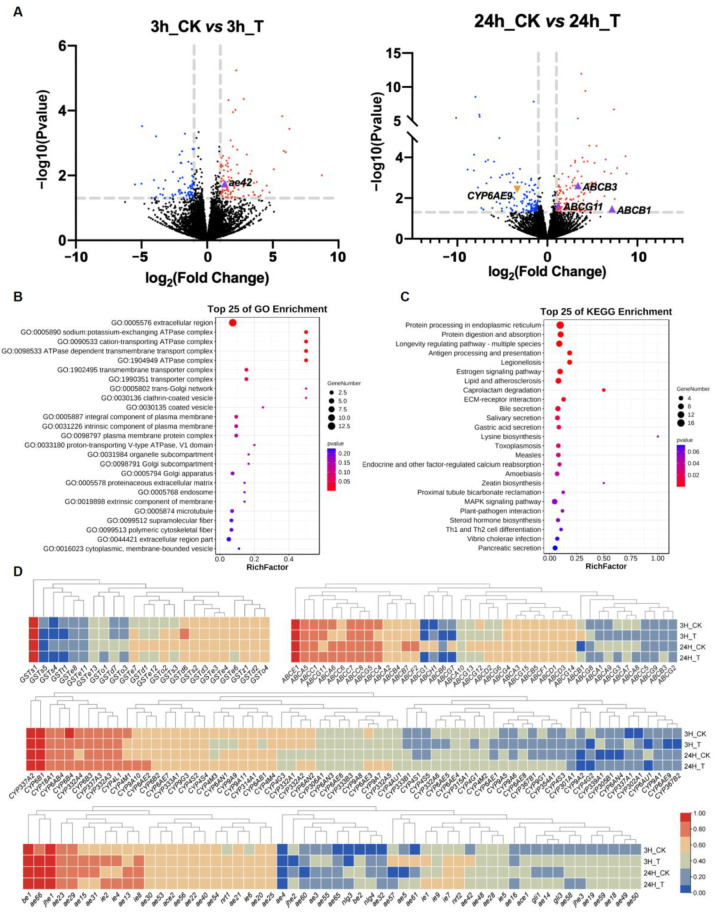
Expression profiles of detoxification genes in *A. pernyi* larvae exposed to coumaphos. (**A**) Plot of significantly differentially expressed genes (DEGs) in the treatment group compared to the control group (3 h, left; 24 h, right; red, up-regulated; blue, down-regulated; black, no difference). (**B**) GO functional enrichment analysis of *A. pernyi* up-regulated genes. (**C**) KEGG functional enrichment analysis of *A. pernyi* up-regulated genes. (**D**) Transcription expression heatmap of detoxification genes induced by coumaphos in *A. pernyi* larvae. CK, control; T, coumaphos treatment.

Based on heatmap analysis, we found that 190 detoxification genes (24 GSTs, 42 ABCs, 68 CYPs, and 56 COEs) were expressed in the larval fat body of the fifth instar (Figure 6D). However, only the *GSTs1* gene from the GST family, the *ABCE1* gene from the ABC family, two genes from the CYP family (*CYP337A2*, and *CYP6B1*), and three genes from the COE family (*ae67*, *be1*, and *jhe3*) were highly expressed in larvae exposed to coumaphos.

The differentially expressed genes (DEGs) were further obtained to examine the transcriptomic alterations caused by the coumaphos treatment. We observed increased expression of 94 genes and decreased expression of 69 genes at 3 h after coumaphos treatment, as well as increased expression of 121 genes and decreased expression of 138 genes at 24 h after treatment (Figure 6A; Supplemental Table S3). GO functional enrichment analysis of the up-regulated genes revealed that there were significant alterations between the coumaphos treatment group and the control group in the pathways related to ATPase complex function, as well as the transporter complex (Figure 6B). KEGG functional enrichment analysis of the up-regulated genes revealed that protein processing in the endoplasmic reticulum was the most affected pathway differing between the coumaphos treatment and control groups. Further, there were significant alterations in the pathways for the longevity regulating pathway, as well as the antigen processing and presentation pathway (Figure 6C). Meanwhile, we also conducted GO and KEGG functional enrichment analysis on all DEGs in the treatment group compared to the control group, and the results were consistent with the study that only included up-regulated genes (Figure S2). 

Finally, we focused on screening genes related to detoxification in the DEGs, and only five genes were found. The *ae43* gene belonging to the COE family was significantly up-regulated in the detoxification gene families after 3 h of coumaphos treatment, whereas *CYP6AE9* belonging to the CYP family was significantly down-regulated, and *ABCB1, ABCB3, and ABCG11* genes belonging to the ABC family were significantly up-regulated after 24 h of treatment. 

## 3. Discussion

In this study, for the first time, we characterized 281 detoxification genes of *A. pernyi* that were irregularly located on 46 chromosomes and may be involved in detoxifying a range of insecticide chemicals. Based on the phylogenetic tree and HMMER searches, these detoxification genes were grouped into four families, consisting of 32 GSTs, 48 ABCs, 104 CYPs, and 97 COEs. According to the functional domains and conserved motif analysis, these detoxification genes exhibited apparent differences in four families. To study how significant detoxification genes in the fat body of *A. pernyi* larvae respond to coumaphos, a transcriptome analysis was performed. The larvae at day 5 of the fifth instar were submerged in coumaphos solution at a mass concentration of 0.025% for 10 s as the treatment group. Ultrapure water was used for the control group. Compared the treatment and control groups, we discovered that the DEGs *ae43*, *ABCB1*, *ABCB3*, and *ABCG11* can be used as potential target genes for further study to explore the underlying molecular mechanisms of detoxification genes involved in resistance towards insecticides in *A. pernyi*. The ABC family can be regarded as an important pathway in the ongoing study of *A. pernyi* resistance. 

We found that the two silkworms *A. pernyi* and *B. mori* have fewer detoxification genes when compared to four nocturnal lepidopteran pests. Several biological characteristics of silkworms may provide clues related to this finding. First, the two silkworms have overly specialized feeding patterns, compared to the four nocturnal pests with various feeding habits and voracious appetites [1,10,11,12]. Rarely do silkworms undergo adaptative changes and subsequent selection of gene expansions due to human domestication. In addition, silkworms lack extensive migratory ability and are rarely exposed to pesticides and plant allelochemicals compared to the other four pests, which may partly limit the expansion of detoxification genes [10,11,12]. Their evolution rate of detoxification-related genes is slower than that of the four pests, and even slightly degenerates. Due to the significant economic value of the two silkworms, no artificial insecticides or pheromones were used on them; hence, there was no selective pressure on evolution. The safe concentration (0.025%) of coumaphos for oak silkworm was employed in this study, under which just the parasitic fly larvae were killed. This also can explain why there are less significant differentially expressed genes in the transcriptional profile between the control and treatment groups. However, with the help of this research, we can more precisely identify the detoxification genes that respond to the coumaphos treatment.

To understand how significant detoxification genes of *A. pernyi* respond to coumaphos, we performed transcriptome research on the fat body treated with coumaphos at two time points. Our study revealed that only five genes (*ae43*, *CYP6AE9*, *ABCB1*, *ABCB3*, and *ABCG11*) were significantly regulated in the detoxification gene families between the control and treatment groups. The up-regulation of *ABCB1, ABCB3,* and *ABCG11* was consistent with previous studies, which reported the detoxification of ABCB and ABCG families in diverse species in response to various types of insecticide [33,34]. The members of the CYP6 subfamily were likewise down-regulated in the oriental fruit moth (*Grapholita molesta*) after 24 h of exposure to three insecticides (malation, deltamethrin, and chlorantraniliprole), demonstrating that the expression of these genes may be inhibited by insecticides over time [35]. In general, *ae43*, *ABCB1*, *ABCB3*, and *ABCG11* genes can be employed as prospective targets of lepidopteran pests for further research. Based on GO enrichment function analysis, the top four affected pathways were all related to the ATPase complex. Therefore, we speculated that the ABC family can be regarded as an essential pathway in the follow-up investigation of *A. pernyi* detoxification ability.

GSTs are vital detoxification enzymes involved in the protection against organophosphorus insecticides in insects [36,37]. Recent studies have disclosed the role of specific GST genes in insecticide detoxification in the Lepidoptera *Cydia pomonella* [38]. Elevated GST activities in *A. pernyi* fat body have been associated with the detoxification of coumaphos [9], as previously observed in most insects [39]. However, we did not observe the significant up-regulation of GST genes in *A. pernyi* fat body when the larvae were exposed to coumaphos under a safe concentration. This discrepancy might be explained by the low safety concentration we utilized, which did not result in the lethal dosage for the oak silkworms. In future trials, we will use higher concentrations of coumaphos or other chemicals that affect GSTs.

In the honey bee, *Apis mellifera*, the up-regulation of *CYP9Q3* plays a key role in the detoxification of coumaphos [40,41]. However, we did not find its homolog in the genomes of *A. pernyi* and *B. mori*. This indicates a difference in detoxification mechanisms between silkworms and bees exposed to coumaphos. Further examination of our data revealed a large number of DEGs, the majority of which were unable to distinguish significantly (*p* > 0.05) between the control and treatment groups. The safe concentration of coumaphos for oak silkworm might be responsible for this. *A. pernyi* grows naturally in the wild, and it is challenging to synchronize its individual development. Moreover, the individual response degree will also differ significantly upon treatment with coumaphos. In the follow-up investigation, we will also pay appropriate attention to the genes with large multiples of difference to uncover any potential detoxification-related target genes of lepidopteran insects. 

## 4. Materials and Methods

### 4.1. Identification of A. pernyi Detoxification Genes

To search for the putative detoxification genes of *A. pernyi*, the GST, ABC transporter, CYP, and COE protein sequences of the domestic silkworm *B. mori* [14] were downloaded from GenBank (ncbi.nlm.nih.gov/, accessed on 29 March 2023) and used as queries to perform local TBLASTN and BLASTP using the BLAST^+^ v2.10.1 tool [42] against *A. pernyi* genomic data [19], with an E-value threshold of 10^−6^. The genomic information for *A. pernyi* was downloaded from Genome Warehouse (GWH) in the National Genomics Data Center (NGDC), Beijing Institute of Genomics (BIG), Chinese Academy of Sciences, with accession number GWHABGR00000000 (https://ngdc.cncb.ac.cn/gwh/, accessed on 20 January 2023) [19]. Potential detoxification genes were further evaluated by HMMER v3.3.2 [43] using a search against the Pfam database (pfam.xfam.org, accessed on 22 Febrary 2023) [44], and the genes with a conserved essential for each gene family were extracted. The theoretical isoelectric point (pI) and molecular weight (MW) were computed using Expasy (https://www.expasy.org/, accessed on 22 Febrary 2023) [45]. 

### 4.2. Phylogenetic Reconstruction

All of the amino acid sequences for the detoxification genes from *A. pernyi* and *B. mori* were aligned using Clustal Omega (https://www.ebi.ac.uk/Tools/msa/clustalo/, accessed on 26 Febrary 2023) [46]. Using the alignment data of the known genes, each identified gene was manually examined and corrected as necessary. The phylogenetic trees were reconstructed with MEGA version X using the neighbor-joining method [47,48], with Poisson correction of distances and boot-strap replications set at 1000. The obtained phylogenetic trees were opened and visualized with Interactive Tree Of Life (iTOL; https://itol.embl.de/, accessed on 26 Febrary 2023) [49].

### 4.3. Analysis of the Conserved Domains and Motifs

The amino acid sequences of the identified detoxification genes of *A. pernyi* were used for subsequent analysis of the conserved domains and motifs. We utilized the Conserved Domain Database of the National Center for Biotechnology Information (NCBI; https://www.ncbi.nlm.nih.gov/Structure/bwrpsb/bwrpsb.cgi, accessed on 26 Febrary 2023) website [50] to determine the conserved domain by analyzing the amino acid sequences of all candidate genes. Protein motifs were determined by Multiple Em for Motif Elicitation (MEME; https://meme-suite.org/meme/tools/meme, accessed on 26 Febrary 2023) [28]. The number of motifs was set to 5, and other parameters were the default parameters. The visualization of domains and motifs was developed using TBtools [51].

### 4.4. RNA-Seq Sample Collection and Illumina Sequencing

The strain Jiaolan of *A. pernyi* was chosen because its complete genome sequence has been published [19] and is more suitable for analyzing gene expression patterns. The strain is maintained at the Oak Silkworm Base of the Department of Sericulture, Shenyang Agricultural University, Liaoning, China. The larvae were reared on the oak tree (*Quercus*) in the field from early May to late June under natural conditions, with a protective net to repel birds. To prevent alterations in the detoxification genes caused by the rhythm, twenty-four larvae on day 5 of the fifth instar were selected in the early morning and divided into two groups, the control and treatment groups. The larvae of the treatment group were submerged in coumaphos solution (trade name Miecanying 4, kindly provided by Liaoning Fenghuang Silkworm Medicine Plant, Fengcheng, China) at a mass concentration of 0.025% for 10 s according to the manufacturer’s protocol. Ultrapure water was used as the treatment for the control group. The treated larvae were still placed on the *Quercus* tree for rearing. After the larvae had been treated for 3 and 24 h [9], the fat bodies were carefully collected, and total RNA was isolated using TRIzol (Beijing Sinogene, China). For sampling, four individuals without significant differences in body weight between the two groups were selected. Personal Biotechnology Co., Ltd., in Nanjing, China completed the library preparation and sequencing of samples. The cDNA library was sequenced on the Illumina HiSeq 2500 sequencing platform using the paired-end technique (2 × 150 bp reads). The sequenced fragments had an average length of 380 base pairs. High-quality clean reads were obtained by the removal of adapters and poor-quality sequences before mapping. Clean reads from each sample were mapped to the *A. pernyi* genome [19] (accession number GWHABGR00000000; available at bigd.big.ac.cn/gwh/, accessed on 20 January 2023) using HISAT2 software v2.2.1 [52], and then HTSeq was used to count the reads that were mapped and calculate the FPKM (fragments per kilobase of exon per million fragments mapped) value of each gene [53]. N50 and mean lengths of the transcripts associated with each sample were calculated. High-quality samples, sequences, and assemblies were indicated by the values for N50 length and mean length for the samples. The Deseq2 software v1.30.0 was used to perform DEG analysis at the threshold of the absolute value of log_2_(Fold Change) > 1 and *p* value < 0.05. The visualization was processed by using R packages [54].

## 5. Conclusions

This study provided the first overview of the detoxification gene set for wild silkworms, which include ~3400 species of Saturniidae and have significant economic importance due to their exceptional-quality fibers and aesthetic and edible values. In the genome of *A. pernyi*, we identified 281 detoxification genes (32 GSTs, 48 ABCs, 104 CYPs, and 97 COEs) with an uneven distribution over 46 chromosomes. Our findings indicated that, except for the ABC family, the semi-domesticated insect *A. pernyi* had a higher number of detoxification genes than the Lepidoptera model *B. mori*. The transcriptome sequencing of the fat body of *A. pernyi* larvae also provided a new perspective for us in searching for potential genes related to the detoxification of Lepidopteran insects. Further studies involving RNA interference or gene knockout of potential target genes would determine their contribution to the detoxification of coumaphos. Such a deeper understanding through genomics and transcriptomics will lay a foundation for exploring the underlying molecular mechanisms of detoxification genes involved in resistance towards insecticides in *A. pernyi*.

## Figures and Tables

**Table 1:** Comparison of the number of detoxification genes from six insect species. “NA” denotes ABC transporter genes that were not shown in the literature.

Species	GSTs	ABCs	CYPs	COEs	Total	References
*Antheraea pernyi*	32	48	104	97	281	This study
*Bombyx mori*	23	52	83	87	245	[14]
*Spodoptera litura*	47	54	138	110	349	[10]
*Spodoptera frugiperda* (corn/rice strain)	46/45	NA/NA	117/135	93/90	256/270	[12]
*Helicoverpa armigera*	42	54	114	97	307	[11]
*Helicoverpa zea*	40	54	108	93	295	[11]

## Data Availability

Data are contained within the article or Supplementary Material.

## Supplementary Materials

The following supporting information can be downloaded at https://www.mdpi.com/article/10.3390/ijms24119775/s1.
