# Supplementary material for: Genome-Wide Identification of Detoxification Genes in Wild Silkworm Antheraea pernyi and Transcriptional Response to Coumaphos
Source: Int J Mol Sci. 2023 Jun 5;24(11):9775. doi: 10.3390/ijms24119775 (PMC10253597; doi:10.3390/ijms24119775)
Supplement: Supplementary file 1 [file ijms-24-09775-s001.zip › Figure S1.pdf]

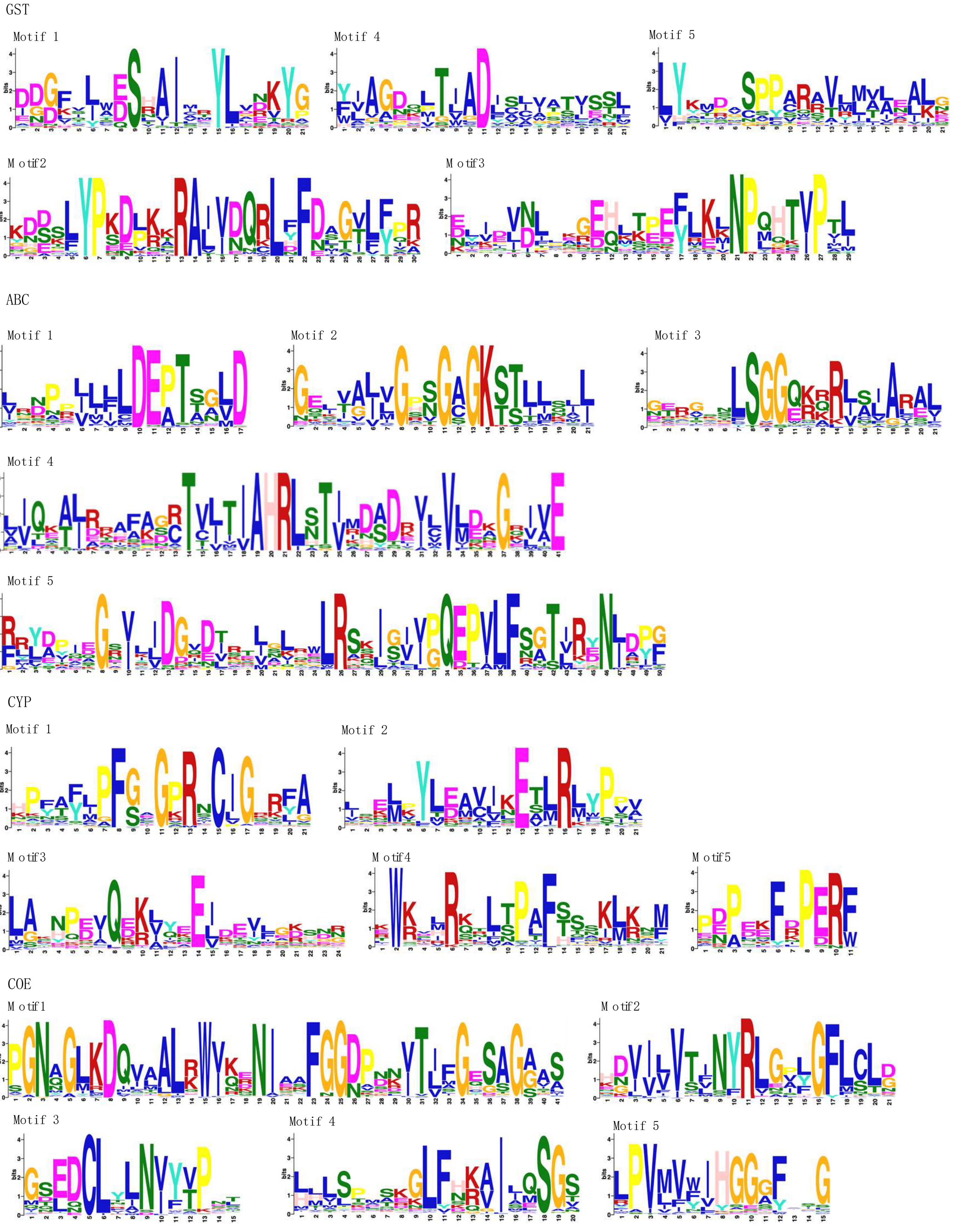

Figure S1 Sequence logos for conserved motifs in *A. pernyi* detoxification genes.
The results produced by multiple expectation for motif elicitation (MEME) program. The height of different amino acids represents repeatability. The scale bar at the bottom indicates the length of the motif protein sequence.
