# Supplementary figures and images for: Genome-Wide Identification of Detoxification Genes in Wild Silkworm Antheraea pernyi and Transcriptional Response to Coumaphos
Source: Int J Mol Sci. 2023 Jun 5;24(11):9775. doi: 10.3390/ijms24119775 (PMC10253597; doi:10.3390/ijms24119775)

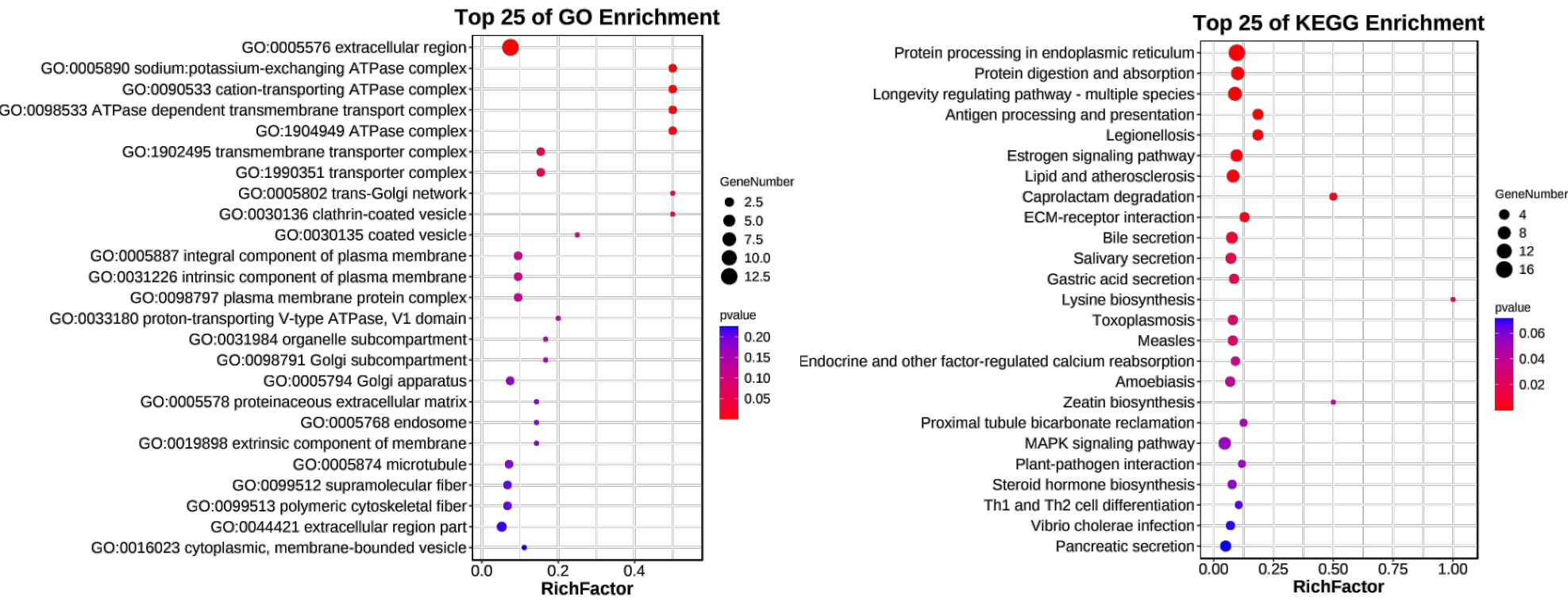

Supplement: Supplementary file 1 [file ijms-24-09775-s001.zip › Figure S2.pdf]
